# Supplementary material for: From sequence to enzyme mechanism using multi-label machine learning
Source: BMC Bioinformatics. 2014 May 19;15:150. doi: 10.1186/1471-2105-15-150 (PMC4229970; doi:10.1186/1471-2105-15-150)
Supplement: Additional file 2 — Java code of ml2db. Additional file ml2db_code.tar.gz contains the Java source code to run the multi-label machine learning experiments and save the results to database. The code’s Javadoc is included. [file 1471-2105-15-150-S2.zip › additional file 2/ml2db/ml2db/doc/mulan/classifier/transformation/LabelPowerset.html]

LabelPowerset


JavaScript is disabled on your browser.


- Overview
- Package
- Class
- Use
- Tree
- Deprecated
- Index
- Help

- Prev Class
- Next Class

- Frames
- No Frames

- All Classes

- Summary:
- Nested |
- Field |
- Constr |
- Method

- Detail:
- Field |
- Constr |
- Method


mulan.classifier.transformation

## Class LabelPowerset

- java.lang.Object
- - mulan.classifier.MultiLabelLearnerBase
  - - mulan.classifier.transformation.TransformationBasedMultiLabelLearner
    - - mulan.classifier.transformation.LabelPowerset

- All Implemented Interfaces:
  :   java.io.Serializable, MultiLabelLearner, weka.core.TechnicalInformationHandler

  Direct Known Subclasses:
  :   LabelsetPruning

  ---

    

  ```
  public class LabelPowerset
  extends TransformationBasedMultiLabelLearner
  ```

  Class that implements a label powerset classifier

  Version:
  :   2012.02.27

  Author:
  :   Grigorios Tsoumakas, Robert Friberg

  See Also:
  :   Serialized Form

- - ### Constructor Summary

    Constructors

    Constructor and Description

    `LabelPowerset(weka.classifiers.Classifier classifier)`

    Conststructor that initializes the learner with a base classifier
  - ### Method Summary

    Methods

    | Modifier and Type | Method and Description |
    | --- | --- |
    | `void` | `setConfidenceCalculationMethod(int method)` Sets the method of calculating probabilities for each label |
    | `void` | `setMakePredictionsBasedOnConfidences(boolean value)` Sets a threshold for obtaining the bipartition |
    | `void` | `setSeed(int s)` Setting a seed for random selection in case of ties during prediction |
    | `void` | `setThreshold(double t)` The threshold for obtaining the bipartition from probabilities |

    - ### Methods inherited from class mulan.classifier.transformation.TransformationBasedMultiLabelLearner

      `getBaseClassifier, getTechnicalInformation, globalInfo`
    - ### Methods inherited from class mulan.classifier.MultiLabelLearnerBase

      `build, getDebug, isUpdatable, makeCopy, makePrediction, setDebug`
    - ### Methods inherited from class java.lang.Object

      `equals, getClass, hashCode, notify, notifyAll, toString, wait, wait, wait`

- - ### Constructor Detail


    - #### LabelPowerset

      ```
      public LabelPowerset(weka.classifiers.Classifier classifier)
      ```

      Conststructor that initializes the learner with a base classifier

      Parameters:
      :   `classifier` - the base single-label classification algorithm
  - ### Method Detail


    - #### setMakePredictionsBasedOnConfidences

      ```
      public void setMakePredictionsBasedOnConfidences(boolean value)
      ```

      Sets a threshold for obtaining the bipartition

      Parameters:
      :   `value` - the threshold's value


    - #### setSeed

      ```
      public void setSeed(int s)
      ```

      Setting a seed for random selection in case of ties during prediction

      Parameters:
      :   `s` - the seed


    - #### setThreshold

      ```
      public void setThreshold(double t)
      ```

      The threshold for obtaining the bipartition from probabilities

      Parameters:
      :   `t` -


    - #### setConfidenceCalculationMethod

      ```
      public void setConfidenceCalculationMethod(int method)
      ```

      Sets the method of calculating probabilities for each label

      Parameters:
      :   `method` -


- Overview
- Package
- Class
- Use
- Tree
- Deprecated
- Index
- Help

- Prev Class
- Next Class

- Frames
- No Frames

- All Classes

- Summary:
- Nested |
- Field |
- Constr |
- Method

- Detail:
- Field |
- Constr |
- Method
